# Nicotinic Antagonist UFR2709 Inhibits Nicotine Reward and Decreases Anxiety in Zebrafish

**DOI:** 10.3390/molecules25132998

**Published:** 2020-06-30

**Authors:** Franco Viscarra, Juan González-Gutierrez, Erica Esparza, Carla Figueroa, Pablo Paillali, Martin Hödar-Salazar, Camilo Cespedes, Gabriel Quiroz, Ramón Sotomayor-Zárate, Miguel Reyes-Parada, Isabel Bermúdez, Patricio Iturriaga-Vásquez

**Affiliations:** 1 Laboratorio de Síntesis Orgánica y Farmacología Molecular, Departamento de Ciencias Químicas y Recursos Naturales, Facultad de Ingeniería y Ciencias, Universidad de la Frontera, Temuco 4811230, Chile; f.viscarra01@ufromail.cl (F.V.); e.esparza01@ufromail.cl (E.E.); c.figueroa17@ufromail.cl (C.F.); p.paillali01@ufromail.cl (P.P.); m.hodar01@ufromail.cl (M.H.-S.); c.cespedes01@ufromail.cl (C.C.); 2 Programa de Doctorado en Química, Facultad de Ciencias, Universidad de Chile, Santiago 7800003, Chile; jpgonzalezg@ug.uchile.cl; 3 Programa de Doctorado en Farmacología, Facultad de Ciencias Químicas y Farmacéuticas, Universidad de Chile, Santiago 8380494, Chile; gquirozn@ciq.uchile.cl; 4 Laboratorio de Neuroquímica y Neurofarmacología, Centro de Neurobiología y Fisiopatología Integrativa (CENFI), Instituto de Fisiología, Facultad de Ciencias, Universidad de Valparaíso, Valparaíso 2360102, Chile; ramon.sotomayor@uv.cl; 5 Centro de Investigación Biomédica y Aplicada (CIBAP), Escuela de Medicina, Facultad de Ciencias Médicas, Universidad de Santiago de Chile, Santiago 9170022, Chile; miguel.reyes@usach.cl; 6 Facultad de Ciencias de la Salud, Universidad Autónoma de Chile, Talca 3467987 Chile; 7 Department of Biological & Medical Sciences, Faculty of Health & Life Sciences, Oxford Brookes University, Oxford OX3 0BP, UK; 8 Center of Excellence in Biotechnology Research Applied to the Environment, Universidad de La Frontera, Temuco 4811230, Chile

**Keywords:** conditioned place preference (CPP), novel tank test (NTT), nicotinic receptor, anxiety, reward, zebrafish

## Abstract

Zebrafish is becoming a popular animal model in neuropharmacology and drug discovery, mainly due to its ease of handling and low costs involved in maintenance and experimental work. This animal displays a series of complex behaviours that makes it useful for assessing the effects of psychoactive drugs. Here, adult zebrafish were used for assessment of the anxiolytic and anti-addictive properties of UFR2709, a nicotinic receptor (nAChR) antagonist, using two behavioural paradigms to test for addiction, the novel tank diving test to assess anxiety and the conditioned place preference (CPP). Furthermore, the expression of nAChR subunits α4 and α7 was measured in the zebrafish brain. The results show that UFR2709 exhibits an anxiolytic effect on zebrafish and blocks the effect evoked by nicotine on CPP. Moreover, UFR2709 significantly decreased the expression of α4 nicotinic receptor subunit. This indicates that UFR2709 might be a useful drug for the treatment of nicotine addiction.

## 1. Introduction

Zebrafish (*Danio rerio*) has been used as a model organism in several fields of research for the past 40 years. This animal model displays relevant traits that makes it a very powerful tool: fast development, high fecundity that provides a large number of individuals, cheap maintenance, and ease of manipulation [1]. Zebrafish also exhibits a variety of complex brain-related behaviours that are believed to parse human behaviours such as learning, attention, memory, aggression, reward, anxiety, and stress [2,3,4,5,6,7,8,9]. Among these, anxiety [10,11,12] and reward [13,14] are the most relevant ones for the characterisation of substances of abuse. Furthermore, the zebrafish neurochemical and neuroanatomical systems share many similarities with mammals [15,16,17] and display analogous sensitivity to a wide spectrum of drugs that act on the central nervous system (CNS) [18], including drugs of abuse.

In mammals, drugs of abuse generate addiction by activating the mesolimbic reward pathway, which is a circuit of dopaminergic neurons going from the ventral tegmental area to the nucleus accumbens [19,20]. Although the brain of the zebrafish lacks these structures, there is evidence for dopaminergic response in the telencephalon of teleosts, which resembles those of the mammalian mesolimbic system [21], and putative homologous structures has been identified in the posterior tubercle of zebrafish that may exert the same function [16,22].

Consequently, the reward-motivated behaviour of zebrafish can be used for determining the addictive properties of drugs through the conditioned place preference (CPP) paradigm. In this test, the reinforcing properties of drugs of abuse serve as a conditioning stimulus (primary motivation) which are repeatedly matched with environmental stimuli (conditioned stimulus), making the fish to change its preference to the side where the drug is administered [23]. This paradigm has been successfully applied to zebrafish by other groups to study the effects of a variety of drugs of abuse, including cocaine, nicotine, ethanol, and D-amphetamine [13,24,25,26,27]. In addition, it has been also applied to determine the effect of novel compounds/mechanisms with possible anti-addictive properties [28,29].

The novel tank diving test is one of the preferred paradigms for measuring anxiolytic profiles of drugs on zebrafish. This model utilises the natural trend of zebrafish to seek refuge when exposed to novel environments, similar to the rodent open field test, and so the fish spends longer periods of time at the bottom of the tank when anxious, a behaviour which is reversed when an anxiolytic drug is administered [11,30].

nAChRs are a wide family of ligand-gated channels that are constituted by 5 subunits forming a pore. The heteromeric α4β2 and the homomeric α7 nAChR subtypes are the most relevant ones, among others [31,32]. nAChR are involved in many different behaviours, including memory [33] and addiction [34], and play a pivotal in the anxiolytic activity of nicotine [35]. In relation to gene expression, it has been demonstrated that nicotine administration produces an increment in the mRNA levels of nAChR subunit α7 in mice [36,37], but it has also been observed that nicotine upregulates nAChRs without noticeable changes in mRNA levels [38,39]. In zebrafish it has been reported that after nicotine treatment that induces CPP, no changes in the mRNA levels of the α4 subunit of nAChR were observed, whereas an increase in the α7 subunit nAChR was detected [24,27].

**Figure 1:**
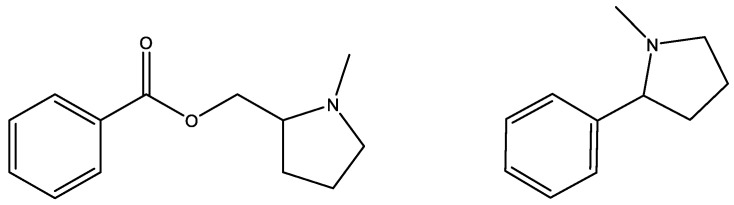
Chemical structure of the nicotinic antagonist UFR2709 and nicotine.

Here, we report that both UFR2709 (Figure 1), a nAChR antagonist that reduces ethanol intake in rats [40], and nicotine exert anxiolytic effects on zebrafish subjected to the novel tank diving test. Furthermore, we show that UFR2709 blocks the effect evoked by nicotine on the CPP paradigm and that both treatments differentially affect the levels of mRNA expression of α4 and α7 nACh receptor subunits in the brain of adult zebrafish. 

## 2. Results

### 2.1. Effects of Nicotine and UFR2709 on the Novel Tank Diving Test (NTT)

**Figure 2:**
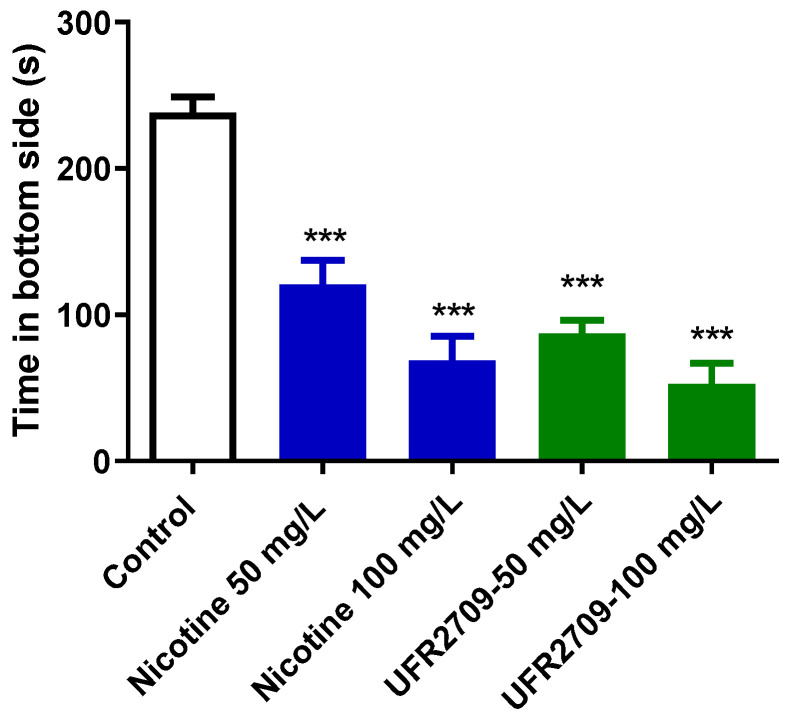
Bottom dwelling time (s) of the swimming zebrafish test using the novel tank test (NTT) on control, nicotine (at 50 and 100 mg/L), and UFR2709 (at 50 and 100 mg/L) groups (*n* = 50). *** *p* < 0.001 using one-way ANOVA.

Adult fish were exposed to nicotine (*n* = 20) or UFR2709 (*n* = 20) for 3 min and were then maintained for another 5 min in a holding tank before testing the swimming behaviour in the test tank for a period of 5 min. Nicotine and UFR2709 were used at two different concentrations, 50 and 100 mg/L. The bottom dwelling time for the control group was 238.4 ± 10.8 s, and this time was significantly reduced to 68.9 ± 16.3 s when the concentration of nicotine was 100 mg/L. Lower levels of nicotine (50 mg/L) produced a smaller but still significant reduction (120.8 ± 22.5 s). UFR2709 at 100 or 50 mg/L also induced a significant and dose-dependent decrease in bottom dwelling time to 52.9 ± 13.8 and 87.0 ± 19.6 s, respectively (Figure 2). These findings indicate that both compounds produce a decrease in the bottom dwelling for novel tank test (NTT).

### 2.2. Effects of Nicotine and UFR2709 on Conditioned Place Preference (CPP)

**Figure 3:**
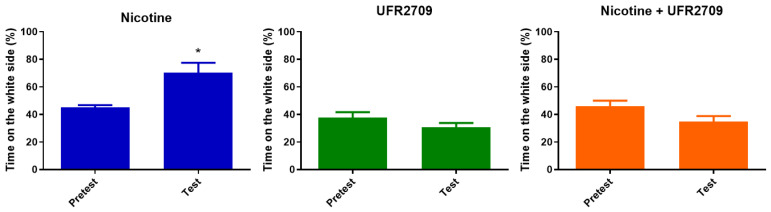
Conditioned place preference. Time spent by the zebrafish on the non-preferred (white) side of the tank (percentage) on pre-test and test days after nicotine at 50 mg/L, UFR2709 at 50 mg/L, or UFR2709 + nicotine (both at 50 mg/L) administration. * *p* > 0.05 using student’s *t*-test (*n* = 24).

The time spent in each compartment (white or brown) on the CPP tank was registered during a period of 10 min. The baseline time and preference place for the free-drug pre-test experiment was estimated using naïve zebrafish. These control experiments indicated the brown compartment as the preferred place (animals spent 60% of the 10 min assay period in this compartment). For the CPP tests, we used 50 mg/L of nicotine (*n* = 8) (we used this concentration to prevent any possible aversive effect at 100 mg/mL) or UFR2709 (*n* = 8). Our CPP studies show that nicotine (50 mg/L) elicited a significant preference for the white compartment (70% of the 10 min period spent in this site versus 40% spent prior to the drug exposure), indicating a conditioning preference induced by nicotine. By contrast, 50 mg/L of UFR2709 did not change the time spent in the non-preferred site (white site) (37% for the pre-test vs. 31% for the test). Interestingly, exposure to 50 mg/L UFR2709 before nicotine treatment (*n* = 8) blocked the conditioning evoked by nicotine (46% for the pre-test vs. 39% for the test after using nicotine/UFR2709) (Figure 3).

### 2.3. Gene Expression of α4 and α7 nACh Receptor Subunits in Adult Zebrafish Brain

**Figure 4:**
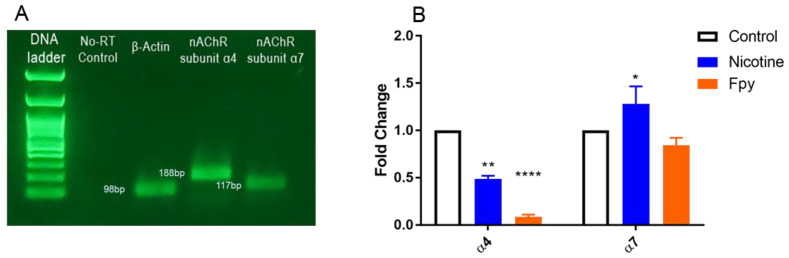
(**A**) Gel electrophoresis of PCR products in control (untreated) animals. Lanes 1: 100 bp DNA ladder; 2: No-RT control; 3: β-Actin; 4: nAChR subunit α4; 5: nAChR subunit α7. (**B**) Expression change of nAChR subunits α4 and α7 after nicotine or UFR2709 treatments (qPCR). * *p* < 0.05, ** *p* < 0.01, and **** *p* < 0.0001 vs. the corresponding control, using student’s *t*-test.

We determined the levels of mRNA expression of α4 and α7 nACh receptor subunits in the brain of adult zebrafish following exposure to UFR2709 or nicotine (under the same condition of CPP experiments) using standard RT-PCR. As shown in Figure 4A, α4 and α7 nicotinic receptor subunits were detected, and the mRNA levels of both subunits were differentially affected by nicotine or UFR2709 (Figure 4B). Thus, the mRNA expression level of the α7 subunit after nicotine treatment was 1.28 fold higher compared to control (*p* < 0.05, student’s *t*-test, *n* = 3). By contrast, levels of α7 mRNA in zebrafish brain after exposure to UFR2709 were not significantly different from control (0.77 versus 1.00). Furthermore, both drug treatments produced a significant decrease in levels of α4 receptor subunit mRNA. α4 expression evoked by nicotine was 0.49 (*p* < 0.01), whereas UFR2709 produced a much stronger decrease of this nAChR subunit, 0.08 fold (*p* < 0.0001), as compared with levels detected in the saline control group.

## 3. Discussion

In this study we used the NTT, a zebrafish model of anxiety, to measure the effects of UFR2709 and nicotine. It has been shown that the extent of the decrease in the period of time the fish spent swimming on the bottom side of the tank is associated with an anxiolytic or pleasant effect [12,31]. Interestingly, both drugs showed the ability to decrease bottom dwelling time, indicating an anxiolytic effect. These results confirm the anxiolytic effects of nicotine, which have been previously reported using the same paradigm [4], and emphasise the usefulness of the novel tank diving test as a good model to assess anxiolytic effects. Furthermore, these apparently controversial results, i.e., the anxiolytic effects after nAChR activation or blockade, are in agreement with previous reports showing that both nAChR agonists and antagonists exhibit anxiolytic-like effects in rodents [41,42,43]. Beyond these considerations, our results demonstrate that UFR2709, a novel nAChR antagonist, acts as an anxiolytic drug in this model.

The CPP is a well-accepted paradigm to evaluate addictive properties of drugs of abuse and the possible anti-addictive effects of novel compounds. Accordingly, nicotine was able to change the preference of the fish through conditioning, spending more time into the non-preferred side (white side) as compared with the pre-test, confirming results described previously [13,24]. On the other hand, UFR2709 did not significantly change the time that the fish spent on the preferred side, indicating that UFR2709 does not produce any conditioning preference. Remarkably, UFR2709, administrated before nicotine, blocked the fish’s place preference stimulated by nicotine alone, an effect that was produced by the administration of UFR2709 before the conditioning sessions. Thus, our results indicate that UFR2709 produces a blockade of the effects of nicotine. In addition, we have recently reported the ability of UFR2709 to decrease ethanol intake in alcohol-preferring rats [40]. Therefore, our current and previous results indicate that this compound might serve to counteract the effects of a relatively wide spectra of addictive compounds and give further support to the notion that nAChR play a pivotal role in drug abuse [44]. 

Using standard RT-PCR, the mRNA of nAChR subunits α4 and α7 were detected in brain homogenates, confirming that these subunits are expressed on the zebrafish brain [45,46]. Moreover, our results indicate that the agonist nicotine and the antagonist UFR2709, after a regime exposure that causes CPP in the case of nicotine, can differentially regulate the mRNA expression of α4 and α7 nAChR subunits. Thus, nicotine treatment produced a decrease in the α4 subunit and an increase in the α7 subunit expression, whereas UFR2709 elicited only a marked decrease in the expression of α4 subunit without modifying the level of the α7 counterpart. This last result is in agreement with previous data showing that UFR2709 displays higher affinity for α4β2 nAChRs than for α7 nAChRs [47]. In our opinion, it is too early to speculate about how these changes in nAChR subunit expression might relate to the effects of both drugs in zebrafish behaviour, but further experiments in this regard are warranted.

## 4. Materials and Methods

### 4.1. Animals

Adult zebrafish, both male and female from TAB-5 wild strain, were used for the behavioural experiments. They were obtained from Dr. M. Allende’s Lab. (Faculty of Sciences, University of Chile) and were kept (5 for each fishbowl in order to avoid stress associated to individual confinement) at 28 °C on a 14:10 light/dark cycle. All experiments were conducted during the light hours. The zebrafish were fed daily with flake tropical fish food. All fish used were drug and experimentally naïve and were used only once. After the trials, the zebrafish were euthanised using ice-cold water.

### 4.2. Drugs

Nicotine tartrate salt was used for the experiments (Sigma-Aldrich). The UFR2709 was synthetised as described previously in the literature [47]. All drugs were dissolved in systems water. The drugs were administered by submerging the fish in either a beaker or a test tank, depending on the experiment, with the dissolved drug at the corresponding concentration. Drug concentrations (50–100 mg/L) were chosen on the basis of literature data for nicotine or UFR2709 in behavioural [4,24], functional [47], or LogP assessments [40].

### 4.3. Novel Tank Test

**Scheme 1:**
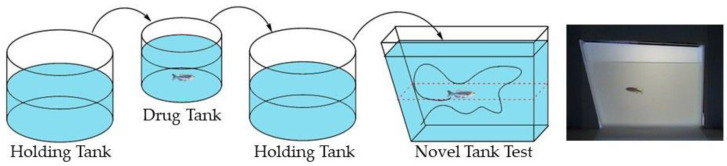
The novel tank diving test (NTT) protocol for to test anxiety behaviour. After acclimation in the first holding tanks, the fish was immersed in the drug tank for 3 min and then transferred to the second holding tank before testing in the tank test for 5 min.

The test tank used was an acrylic trapezoid 14.5 cm in height, 22 cm at the bottom, 27 cm at the top, with a diagonal of 16 cm, 5 cm wide, and filled with 1.6 L of water. During the experiment, one fish (*n* = 10 per drugs at 50 and 100 mg/L plus controls) was placed in a holding tank for 5 min for acclimation, and then it was immersed in another tank with the dissolved drugs (nicotine and UFR2709 at 50 and 100 mg/L), or regular water in the case of the control group, for 3 min. Then, it was transferred to a second holding tank for 5 min and, finally, to the test tank where swimming behaviour was recorded for 5 min (Scheme 1). The whole procedure was done according to previous reports [30]. For recording purposes, a USB webcam was used, and the tank was backlit with a white acrylic screen to provide enough contrast for detection. The swimming trajectories were analysed using Noldus Ethovision XT software (Wageningen, The Netherlands). The novel tank test was divided in two sections using Ethovision software and the time spent at the bottom section was recorded.

### 4.4. Conditioned Place Preference

**Scheme 2:**
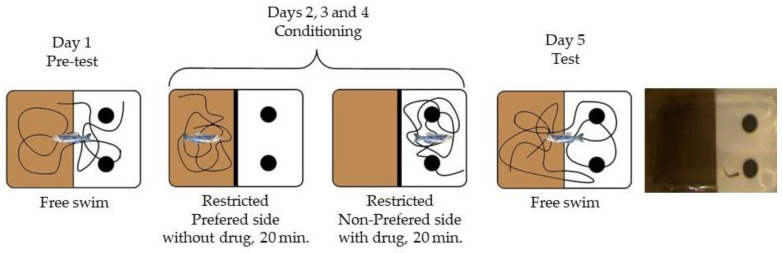
The conditioned place preference (CPP) protocol to test addiction. Day 1 pre-test, the fish swims freely, and the preferred side was selected over 10 min of recording. Day 2, 3, and 4 conditioning, the fish was restricted to the preferred side without drugs for 20 min and was then restricted to the non-preferred side with drugs for 20 min. Day 5 test, the fish swims freely and was recorded for 10 min for the CPP test.

Our test tank was designed according to the specifications of Ninkovic and Bally-Cuif [13]. The tank dimensions were 22 cm length, 16 cm width, and 12.5 cm depth, and it was filled with 2 L of water. Two different cues divided the tank in two halves: the first half was white with two black dots on the bottom of the tank and the second half was darker with a light brown colour. For the recording, the tank was placed in an isolated chamber with white backlight at the bottom and a camera at the top. The experiment was carried out over five days (8 fish were used for each experiment): on the first day, the pre-test; on the second, third, and fourth day, the conditioning; and on the final day, the test. The pre-test consisted of leaving the fish to swim across the tank for 15 min. After an initial 5 min of habituation period in the tank, the baseline preference was measured by recording the swimming behaviour for 10 min. The preferred compartment was defined as the compartment in which a fish spends most time during the 10 min of the pre-test. In the conditioning for the nicotine control group, the zebrafish were first confined to the preferred side for 20 min and then to the non-preferred side while exposed to 50 mg/L nicotine for 20 min. For the UFR2709 group, the same protocol was followed but using UFR2709 50 mg/L in the non-preferred side instead of nicotine. Finally, the UFR2709 + nicotine group received the same conditioning as with nicotine 50 mg/L, but before exposing the zebrafish to nicotine on the non-preferred side, the animal was immersed in a beaker with 50 mg/L UFR2709 for 5 min. On the fifth day, the test was performed in the same way as the pre-test to establish the final preference (Scheme 2). For both the pre-test and the test the recordings were analysed with Noldus Ethovision XT software (Wageningen, The Netherlands).

### 4.5. Reverse Transcription and quantitative Polymerase Chain Reaction (RT-qPCR)

After treatment (under the same condition of CPP experiments), four animals were euthanised, dissected, and their brain homogenised in lysis buffer from a total RNA extraction kit (Geneaid Biotech, New Taipei City, Taiwan) and were considered one sample. Extracted RNA was treated with DNase-I (30 min, 37 °C) to get rid of genomic DNA. cDNA was synthesised from RNA with the ImProm-II Reverse Transcription System (Promega, Madison, WI, USA) using oligo dT primers. cDNA (1 µL) was used for either standard or quantitative PCR (StepOne Real-Time PCR System, Thermo Fisher Scientific, Waltham, Massachusetts, USA) using GoTaq Green Master Mix (Promega, Madison, WI, USA) or HOT FIREPol EvaGreen qPCR Supermix (Solis BioDyne, Tartu, Estonia) respectively. Specific primers were designed using Primer BLAST tool [48] from the zebrafish genome reported in the NCBI database (Table 1). PCR products were visualised on a 1.5% agarose gel. The comparative ΔΔCT method was used for quantification with β-actin as the internal reference gene. β-Actin expression showed no significant variation between control and treatment groups.

### 4.6. Statistical Analysis

The data were analysed using student’s *t*-test or one-way ANOVA, depending on experiment design. The statistical analysis and data visualisation was carried out with GraphPad Prism 8 for Windows. The data shown are presented as the mean ± SEM with *p* < 0.05, *p* < 0.01, *p* < 0.001, and *p* < 0.0001 regarded as statistically significant.

## 5. Conclusions

In the present study, we confirmed the anxiolytic effect of nicotine in zebrafish using the NTT paradigm. Additionally, the addictive properties of nicotine were also confirmed using a CCP paradigm for zebrafish, which is analogous to the model used for rodents. The novel nAChR antagonist UFR2709 showed anxiolytic properties in the NTT and blocked the effect on the CCP evoked by nicotine. In addition, we showed that nAChR subunit gene expression can be differentially regulated by nicotinic agonists and antagonists. In summary, our results indicate that UFR2709 is a novel drug with an attractive pharmacological profile, potentially useful for the treatment of nicotine addiction and/or anxiety, and support the suitability of zebrafish as a neuropharmacological model.

## Figures and Tables

**Table 1:** Sequence of the primers used to study zebrafish mRNA expression.

Gene	Forward Primer	Reverse Primer
β-Actin (NM_131031.1)	CGAACGACCAACCTAAACCTC	ACCTCCCTTTCCAGTTTCCG
nAChR subunit α4 (NM_001048063.1)	CATGCCCATGCGGAAGAAAG	TCGTTCCACTCCTGCTTCAC
nAChR subunit α7 (NM_201219.2)	TGCTGCCTATGGAGTGTGTC	CGTGAGTGAGTGGGTGTCAT

## References

[1] Meyers J.R. (2018). Zebrafish: Development of a Vertebrate Model Organism. Curr. Protoc. Essent. Lab. Tech..

[2] Gerlai R., Lahav M., Guo S., Rosenthal A. (2000). Drinks like a fish: Zebra fish (Danio rerio) as a behavior genetic model to study alcohol effects. Pharmacol. Biochem. Behav..

[3] Egan R.J., Bergner C.L., Hart P.C., Cachat J.M., Canavello P.R., Elegante M.F., Elkhayat S.I., Bartels B.K., Tien A.K., Tien D.H. (2009). Understanding behavioral and physiological phenotypes of stress and anxiety in zebrafish. Behav. Brain Res..

[4] Levin E.D., Bencan Z., Cerutti D.T. (2007). Anxiolytic effects of nicotine in zebrafish. Physiol. Behav..

[5] Swain H.A., Sigstad C., Scalzo F.M. (2004). Effects of dizocilpine (MK-801) on circling behavior, swimming activity, and place preference in zebrafish (Danio rerio). Neurotoxicol. Teratol..

[6] Carvan M.J., Loucks E., Weber D.N., Williams F.E. (2004). Ethanol effects on the developing zebrafish: Neurobehavior and skeletal morphogenesis. Neurotoxicol. Teratol..

[7] Williams F.E., White D., Messer W.S. (2002). A simple spatial alternation task for assessing memory function in zebrafish. Behav. Processes.

[8] Iturriaga-Vásquez P., Osorio F., Riquelme S., Castro S., Herzog R. (2012). Zebrafish: A Model for Behavioral Pharmacology. Farmacol. Chile.

[9] van Staden C., de Brouwer G., Botha T.L., Finger-Baier K., Brand S.J., Wolmarans D. (2020). Dopaminergic and serotonergic modulation of social reward appraisal in zebrafish (Danio rerio) under circumstances of motivational conflict: Towards a screening test for anti-compulsive drug action. Behav. Brain Res..

[10] Bencan Z., Sledge D., Levin E.D. (2009). Buspirone, chlordiazepoxide and diazepam effects in a zebrafish model of anxiety. Pharmacol. Biochem. Behav..

[11] Duarte T., Fontana B.D., Müller T.E., Bertoncello K.T., Canzian J., Rosemberg D.B. (2019). Nicotine prevents anxiety-like behavioral responses in zebrafish. Prog. Neuro-Psychopharmacol. Biol. Psychiatry.

[12] Stewart A., Wu N., Cachat J., Hart P., Gaikwad S., Wong K., Utterback E., Gilder T., Kyzar E., Newman A. (2011). Pharmacological modulation of anxiety-like phenotypes in adult zebrafish behavioral models. Prog. Neuropsychopharmacol. Biol. Psychiatry.

[13] Ninkovic J., Bally-Cuif L. (2006). The zebrafish as a model system for assessing the reinforcing properties of drugs of abuse. Methods.

[14] Faillace M.P., Pisera-Fuster A., Bernabeu R. (2018). Evaluation of the rewarding properties of nicotine and caffeine by implementation of a five-choice conditioned place preference task in zebrafish. Prog. Neuro-Psychopharmacol. Biol. Psychiatry.

[15] Panula P., Chen Y.-C., Priyadarshini M., Kudo H., Semenova S., Sundvik M., Sallinen V. (2010). The comparative neuroanatomy and neurochemistry of zebrafish CNS systems of relevance to human neuropsychiatric diseases. Neurobiol. Dis..

[16] Rink E., Wullimann M.F. (2002). Connections of the ventral telencephalon and tyrosine hydroxylase distribution in the zebrafish brain (Danio rerio) lead to identification of an ascending dopaminergic system in a teleost. Brain Res. Bull..

[17] Saleem S., Kannan R.R. (2018). Zebrafish: An emerging real-time model system to study Alzheimer’s disease and neurospecific drug discovery. Cell Death Discov..

[18] Khan K.M., Collier A.D., Meshalkina D.A., Kysil E.V., Khatsko S.L., Kolesnikova T., Morzherin Y.Y., Warnick J.E., Kalueff A.V., Echevarria D.J. (2017). Zebrafish models in neuropsychopharmacology and CNS drug discovery. Br. J. Pharmacol..

[19] Wise R.A. (1996). Neurobiology of addiction. Curr. Opin. Neurobiol..

[20] Volkow N.D., Wise R.A., Baler R. (2017). The dopamine motive system: Implications for drug and food addiction. Nat. Rev. Neurosci..

[21] Mok E.Y.-M., Munro A. (1998). Effects of dopaminergic drugs on locomotor activity in teleost fish of the genus Oreochromis (Cichlidae): Involvement of the telencephalon. Physiol. Behav..

[22] Rink E., Wullimann M.F. (2001). The teleostean (zebrafish) dopaminergic system ascending to the subpallium (striatum) is located in the basal diencephalon (posterior tuberculum). Brain Res..

[23] Mathur P., Lau B., Guo S. (2011). Conditioned place preference behavior in zebrafish. Nat. Protoc..

[24] Kedikian X., Faillace M.P., Bernabeu R. (2013). Behavioral and Molecular Analysis of Nicotine-Conditioned Place Preference in Zebrafish. PLoS ONE.

[25] Darland T., Dowling J.E. (2001). Behavioral screening for cocaine sensitivity in mutagenized zebrafish. Proc. Natl. Acad. Sci. USA.

[26] Mathur P., Berberoglu M.A., Guo S. (2011). Preference for ethanol in zebrafish following a single exposure. Behav. Brain Res..

[27] Pisera-Fuster A., Rocco L., Faillace M.P., Bernabeu R. (2019). Sensitization-dependent nicotine place preference in the adult zebrafish. Prog. Neuro-Psychopharmacol. Biol. Psychiatry.

[28] Pisera-Fuster A., Faillace M.P., Bernabeu R. (2020). Pre-Exposure to Nicotine with Nocturnal Abstinence Induces Epigenetic Changes that Potentiate Nicotine Preference. Mol. Neurobiol..

[29] Braida D., Ponzoni L., Moretti M., Viani P., Pallavicini M., Bolchi C., Appiani R., Bavo F., Gotti C., Sala M. (2020). Behavioural and pharmacological profiles of zebrafish administrated pyrrolidinyl benzodioxanes and prolinol aryl ethers with high affinity for heteromeric nicotinic acetylcholine receptors. Psychopharmacology (Berl).

[30] Gomez-Molina C., Ortiz-severin J., Osorio F., Quiroz G., Reyes-parada M., Varas R., Moya P.R., Iturriaga-Vásquez P. (2015). Effects of selective α4 β2 Nicotinic acetylcholine receptor (nAChR) ligands on the behaviour of adult zebrafish (Danio rerio) in the novel tank diving task. Rev. Farmacol. Chile.

[31] Gotti C., Zoli M., Clementi F. (2006). Brain nicotinic acetylcholine receptors: Native subtypes and their relevance. Trends Pharmacol. Sci..

[32] Albuquerque E.X., Pereira E.F.R., Alkondon M., Rogers S.W. (2009). Mammalian Nicotinic Acetylcholine Receptors: From Structure to Function. Physiol. Rev..

[33] Galvin V.C., Arnsten A.F.T., Wang M. (2020). Involvement of Nicotinic Receptors in Working Memory Function.

[34] Papke R.L., Brunzell D.H., De Biasi M. (2020). Cholinergic Receptors and Addiction.

[35] Bourbon A., Boyer L., Auquier P., Boucekine M., Barrow V., Lançon C., Fond G. (2019). Anxiolytic consumption is associated with tobacco smoking and severe nicotine dependence. Results from the national French medical students (BOURBON) study. Prog. Neuropsychopharmacol. Biol. Psychiatry.

[36] Ryan R.E., Loiacono R.E. (2001). Nicotine regulates alpha7 nicotinic receptor subunit mRNA: Implications for nicotine dependence. Neuroreport.

[37] Fowler C.D., Lu Q., Johnson P.M., Marks M.J., Kenny P.J. (2011). Habenular α5 nicotinic receptor subunit signalling controls nicotine intake. Nature.

[38] Wonnacott S. (1990). The paradox of nicotinic acetylcholine receptor upregulation by nicotine. Trends Pharmacol. Sci..

[39] Govind A.P., Vezina P., Green W.N. (2009). Nicotine-induced upregulation of nicotinic receptors: Underlying mechanisms and relevance to nicotine addiction. Biochem. Pharmacol..

[40] Quiroz G., Sotomayor-Zárate R., González-Gutierrez J.P., Viscarra F., Moraga F., Bermudez I., Reyes-Parada M., Quintanilla M.E., Lagos D., Rivera-Meza M. (2019). UFR2709, a Nicotinic Acetylcholine Receptor Antagonist, Decreases Ethanol Intake in Alcohol-Preferring Rats. Front. Pharmacol..

[41] Brioni J.D., O’Neill A.B., Kim D.J.B., Decker M.W. (1993). Nicotinic receptor agonists exhibit anxiolytic-like effects on the elevated plus-maze test. Eur. J. Pharmacol..

[42] Targowska-Duda K.M., Budzynska B., Michalak A., Jozwiak K., Biala G., Arias H.R. (2019). 3-Furan-2-yl-N-p-tolyl-acrylamide, a highly selective positive allosteric modulator of α7 nicotinic receptors, produces anxiolytic-like activity in mice. J. Psychopharmacol..

[43] Hall B., Pearson L., Buccafusco J. (2010). Effect of the use-dependent, nicotinic receptor antagonist BTMPS in the forced swim test and elevated plus maze after cocaine discontinuation in rats. Neurosci. Lett..

[44] Fagen Z.M., Mitchum R., Vezina P., McGehee D.S. (2007). Enhanced Nicotinic Receptor Function and Drug Abuse Vulnerability. J. Neurosci..

[45] Zirger J.M., Beattie C.E., McKay D.B., Thomas Boyd R. (2003). Cloning and expression of zebrafish neuronal nicotinic acetylcholine receptors. Gene Expr. Patterns.

[46] Ackerman K.M., Nakkula R., Zirger J.M., Beattie C.E., Boyd R.T. (2009). Cloning and spatiotemporal expression of zebrafish neuronal nicotinic acetylcholine receptor alpha 6 and alpha 4 subunit RNAs. Dev. Dyn..

[47] Faundez-Parraguez M., Farias-Rabelo N., Gonzalez-Gutierrez J.P., Etcheverry-Berrios A., Alzate-Morales J., Adasme-Carreño F., Varas R., Bermudez I., Iturriaga-Vasquez P. (2013). Neonicotinic analogues: Selective antagonists for α4β2 nicotinic acetylcholine receptors. Bioorg. Med. Chem..

[48] Ye J., Coulouris G., Zaretskaya I., Cutcutache I., Rozen S., Madden T.L. (2012). Primer-BLAST: A tool to design target-specific primers for polymerase chain reaction. BMC Bioinform..

